# Bat Coronaviruses and Experimental Infection of Bats, the Philippines

**DOI:** 10.3201/eid1608.100208

**Published:** 2010-08

**Authors:** Shumpei Watanabe, Joseph S. Masangkay, Noriyo Nagata, Shigeru Morikawa, Tetsuya Mizutani, Shuetsu Fukushi, Phillip Alviola, Tsutomu Omatsu, Naoya Ueda, Koichiro Iha, Satoshi Taniguchi, Hikaru Fujii, Shumpei Tsuda, Maiko Endoh, Kentaro Kato, Yukinobu Tohya, Shigeru Kyuwa, Yasuhiro Yoshikawa, Hiroomi Akashi

**Affiliations:** University of Tokyo, Tokyo, Japan (S. Watanabe, N. Ueda, K. Iha, S. Taniguchi, H. Fujii, S. Tsuda, M. Endoh, K. Kato, S. Kyuwa, Y. Yoshikawa, H. Akashi); University of the Philippines Los Baños, Laguna, the Philippines (J.S. Masangkay, P. Alviola); National Institute of Infectious Diseases, Tokyo (N. Nagata, S. Morikawa, T. Mizutani, S. Fukushi, T. Omatsu); Nihon University, Kanagawa, Japan (Y. Tohya)

**Keywords:** Bat coronavirus, severe acute respiratory syndrome, SARS, experimental infection, bats, coronavirus, viruses, the Philippines, zoonoses, research

## Abstract

Fifty-two bats captured during July 2008 in the Philippines were tested by reverse transcription–PCR to detect bat coronavirus (CoV) RNA. The overall prevalence of virus RNA was 55.8%. We found 2 groups of sequences that belonged to group 1 (genus *Alphacoronavirus*) and group 2 (genus *Betacoronavirus*) CoVs. Phylogenetic analysis of the RNA-dependent RNA polymerase gene showed that groups 1 and 2 CoVs were similar to Bat-CoV/China/A515/2005 (95% nt sequence identity) and Bat-CoV/HKU9–1/China/2007 (83% identity), respectively. To propagate group 2 CoVs obtained from a lesser dog-faced fruit bat (*Cynopterus brachyotis*), we administered intestine samples orally to Leschenault rousette bats (*Rousettus leschenaulti*) maintained in our laboratory. After virus replication in the bats was confirmed, an additional passage of the virus was made in Leschenault rousette bats, and bat pathogenesis was investigated. Fruit bats infected with virus did not show clinical signs of infection.

## Abstract 2

Virus-infected fruit bats showed no signs of clinical infection.

Severe acute respiratory syndrome (SARS) coronavirus (SARS-CoV) is a newly emerged zoonotic CoV that caused an international epidemic in 2003. Epidemiologic studies have demonstrated that the first human cases of SARS were caused by CoVs closely related to those found in Himalayan palm civets and raccoon dogs in wildlife markets (*1*). This finding accelerated surveys of CoVs specific for various animals in Southeast Asia to identify reservoirs for SARS-CoV. These survey findings suggested that palm civets and raccoon dogs are an intermediate host of, but not a primary reservoir for, SARS-CoV because of the low prevalence of SARS-like CoVs in these animals (*2*). Moreover, a large variety of novel CoVs in these surveys, including bat SARS–like CoVs, were detected in many bat species in the People’s Republic of China and Hong Kong Special Administrative Region (*3*–*6*).

Phylogenetic analysis of bat CoVs and other known CoVs suggested that the progenitor of SARS-CoV and all other CoVs in other animal hosts originated in bats (*5*,*7*). Recently, bat CoVs in North and South America, Europe, and Africa were also reported (*8*–*12*). Although extensive bat surveys have been conducted, no infectious bat CoVs have been isolated from cell cultures, which hinders characterization of bat CoVs and evaluation of the risks posed by these viruses to public health.

In this study, we detected bat CoVs in the Philippines. We attempted to isolate bat CoVs and virus RNA from cell cultures and from Leschenault rousette bats (*Rousettus leschenaulti*) orally infected with intestinal tissues and contents from a lesser dog-faced fruit bat (*Cynopterus brachyotis*). After infection, clinical signs of infected bats were examined, and pathogenesis in bats was investigated.

## Materials and Methods

### Bat Collection

We obtained 52 bats of 6 species during July 2008 from Diliman and Los Baños, the Philippines, after receiving permission from the government. All captured bats were anesthetized with an intraperitoneal injection (15 mg/kg) of tiletamine and zolazepam (Virbac, Carros, France) and killed by cardiac exsanguination. The experiment was conducted in accordance with the Guidelines for the Care and Use of Laboratory Animals, Graduate School of Agriculture and Life Sciences, University of Tokyo.

### Extraction of RNA and Reverse Transcription

Virus RNA was extracted from samples obtained from field bats and from experimentally infected bats by using an SV Total RNA Isolation System Kit (Promega, Madison, WI, USA) according to the manufacturer’s instructions. Extracted RNA was eluted in 50 µL of RNase-free water. For cDNA synthesis, RNA (5 μL), a random hexamer, and a SuperScript III Kit (Invitrogen, Carlsbad, CA, USA) were used.

### PCR and DNA Sequencing

All cDNA samples obtained from field bats and experimentally infected bats were tested by using conventional and nested PCR. On the basis of previous reports, we used a PCR and a pair of consensus primers specific for a highly conserved region of the RNA-dependent RNA polymerase (RdRp) gene (*13*).

Two microliters of cDNA was added to a 25-μL reaction mixture containing 2× GoTaq PCR Master Mix (Promega) and 0.2 μM of 5′-GGTTGGGACTATCCTAAGTGTGA-3′ (primer 1) and 5′-CCATCATCAGATAGAATCATCATA-3′ (primer 2). The PCR conditions were 2 min at 94°C; 35 cycles for 20 s at 94°C, 30 s at 50°C, and 30 s at 72°C; and 1 min at 72°C. PCR amplicons were gel purified by using NucleoSpin Extract II (Machrey-Nagel, Düren, Germany) and cloned by using a TOPO-TA pCR2.1 Cloning Kit (Invitrogen). Sequencing was performed in an ABI 3130 XL DNA analyzer (Applied Biosystems, Foster City, CA, USA).

On the basis of the sequences obtained, we designed new specific primer pairs for the Bat-CoV/Philippines/Diliman1552G1/2008 sequence (5′-TGATTTCTGCAATGATACTTGGTTC-3′ and 5′-ACTTGATGATCTGTAACAACAATCG-3′) and for the Bat-CoV/Philippines/Diliman1525G2/2008 sequence (5′-TACAACCTACGCTGCAACTC-3′ and 5′-ATGAGTGTGCACAAGTGCTTAG-3′). These primers were used as the inner primer set for the nested PCR after the first PCR was performed with primers 1 and 2. Aliquots (2 µL) of cDNA for primary amplification were added to 2× GoTaq Master Mix (Promega) and primers 1 and 2. Amplification was performed by using 15 cycles at conditions described above. Aliquots (2 µL) of primary amplification products were used for the second PCR with GoTaq Master Mix and the inner primers. The second PCR was performed by using 35 cycles at the conditions described above. PCR products were extracted from gels by using NucleoSpin Extract II and subjected to direct sequencing or TA cloning.

### Bat Samples

Leschenault rousette bats were obtained from zoos in Japan. Seven bats were randomly selected for the experiments. In each experiment, 2 bats were placed in a negative-pressure isolator. One additional bat was kept in a separate isolator as a control. A sample of large intestine from a lesser dog-faced fruit bat (*C. brachyotis*) was homogenized in a sterile mortar. After low-speed centrifugation (2,000 × *g* for 10 min), the supernatant was used for oral infection. Experimentally infected bats were examined daily for clinical signs of infection. Fecal specimens were obtained from a clean translucent plastic sheet spread along the bottom of the cage. All bats were killed after being anesthetized with diethyl ether, and organs (liver, kidney, spleen, lung, brain, and intestine) and serum samples were obtained.

### Detection of Virus mRNA in Bats

To determine membrane, nucleocapsid, nonstructural (Ns)7a, Ns7b, and Ns7c protein nucleotide sequences, we conducted PCR and DNA sequencing in the same manner as for determination of partial RdRp nucleotide sequence described above by using the HKU9-Leader42–64 primer (5′-CCGTTTCGTCTTGTACGAATCAC-3′) and the 3siteAd20T primer (5′-CTGATCTAGAGGTACCGGATCCTTTTTTTTTTTTTTTTTTTT-3′). To detect virus mRNA, we conducted reverse transcription–PCR (RT-PCR) by using 2 primer sets: HKU9-Leader42–64 and N468–448r (5′-GTTACGTGTGCCCATGTCACC-3′) and HKU9-Leader42–64 and Ns7a440–420r (5′-CAAGCCACAACAACATTAGG-3′).

### Quantitative Real-Time RT-PCR

cDNA synthesis was performed by using 0.5 μL total RNA and the PrimeScript RT Reagent Kit (TaKaRa, Shiga, Japan) according to the manufacturer’s protocol. Virus RNA was quantified by using Power SYBR Green PCR Master Mix (Applied Biosystems) with 2 μL of reverse-transcribed cDNA. Quantitative real-time PCR was performed by using the Thermal Cycler Dice System (TaKaRa). The temperature program consisted of an initial denaturation at 95°C for 10 min, followed by 40 cycles at 95°C for 15 s and 60°C for 1 min. The primer pair for the real-time PCR was designed on the basis of the partial RdRp sequences of Bat-CoV/Philippines/Diliman1525G2/2008; primers used were 5′-TCCTAAGTGTGATAGAGCTATGCC-3′ and 5′-GTGCACACTCATTTGCTAACCG-3′. In each experiment, 10-fold serial dilutions of plasmid DNA containing the partial RdRp gene were tested in duplicate to establish a standard curve for calculating the relative amount of RNA in each sample. All samples were analyzed at least 3 times. To confirm the specificity of each PCR product, we conducted a melting curve analysis immediately after the amplification phase of each PCR. The amount of RNA in each sample was expressed as the average value (copy number per weight [milligrams] of sample).

## Results

### Virus Detected

During July 2008, a total of 52 bats were obtained at 3 locations in Diliman and 1 location in Los Baños, the Philippines (Table 1). RT-PCRs for a 440-bp fragment in the RdRp gene of CoVs were performed for large intestine samples, including intestinal contents; 9 (17.3%) of 52 bats were positive. Differences in the 440-nt sequence in the RdRp region were determined after TA cloning of the 9 positive samples. Sequences indicated that the 2 groups of sequences obtained belonged to group 1 CoV (genus *Alphacoronavirus*) (n = 4) and group 2 CoV (genus *Betacoronavirus*) (n = 5).

**Table 1:** Prevalence of coronavirus in bats, the Philippines

Sampling site	Common name (species)	No. intestine samples tested	No. positive (group 1)	No. positive (group 2)
Los Baños	Lesser dog-faced fruit bat (*Cynopterus brachyotis*)	4	0	2
	Cave nectar bat (*Eonycteris spelaea*)	3	0	2
	Greater musky fruit bat (*Ptenochirus jagori*)	14	0	11
Diliman (site A)	Lesser dog-faced fruit bat (*C. brachyotis*)	1	0	1
	Cave nectar bat (*E. spelaea*)	1	0	1
	Greater musky fruit bat (*P. jagori*)	1	0	0
Diliman (site B)	Cave nectar bat (*E. spelaea)*	1	0	1
	Java pipistrelle bat (*Pipistrellus javanicas*)	3	0	0
	Lesser Asiatic yellow bat (*Scotphilus kuhlii*)	4	4	0
Diliman (site C)	Lesser dog-faced fruit bat (*C. brachyotis*)	18	0	6
	Greater musky fruit bat (*P. jagori*)	1	0	0
	Geoffroy rousette bat (*Rousettus amplexicaudatus*)	1	0	1
Total		52	4	25

A 440-bp consensus nt sequence of the group 1 CoV was obtained on the basis of alignment of 4 group 1 CoV sequences detected (>98% nt identity with each other) and deposited in GenBank as Bat-CoV/Philippines/Diliman1552G1/2008 (DNA Database of Japan [DDBJ] accession no. AB539080). BLAST (www.ncbi.nlm.nih.gov/BLAST) search findings of GenBank indicated that the partial RdRp sequence was most similar to that of Bat-CoV/China/A515/2005 (95% nt identity).

**Figure 1:**
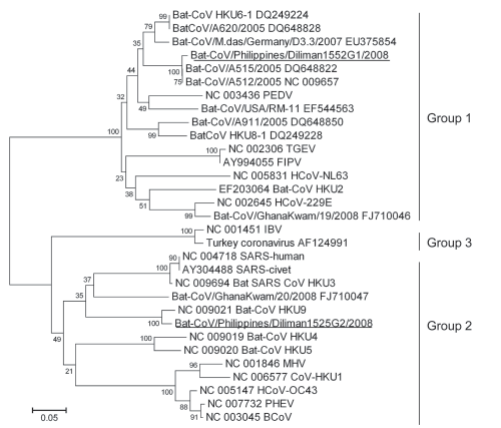
Phylogenetic tree based on deduced amino acid sequences of partial RNA-dependent RNA polymerase of coronaviruses (CoVs), the Philippines. The 2 new viruses detected in this study are underlined. Percentage of replicate trees in which the associated taxa clustered in the bootstrap test (1,000 replicates) is shown next to the branches. The tree is drawn to scale, with branch lengths in the same units as those of the evolutionary distances used to infer the phylogenetic tree. Evolutionary distances were computed by using the Poisson correction method and are shown as number of amino acid substitutions per site. All positions containing gaps and missing data were eliminated from the dataset. The final dataset included 120 positions. Phylogenetic analyses were conducted in MEGA4 (*14*). Coronaviruses used for comparisons and their GenBank accession numbers are human coronavirus (HCoV) 229E (HCoV-229E) (NC_002645), porcine epidemic diarrhea virus (PEDV) (NC_003436), transmissible gastroenteritis virus (TGEV) (NC_002306), feline infectious peritonitis virus (FIPV) (AY994055), human coronavirus NL63 (HCoV-NL63) (NC_005831), bat-CoV/A512/2005 (NC_009657), bat-CoV/A515/2005 (DQ648822), bat-CoV/A620/2005 (DQ648828), bat-CoV/A911/2005 (DQ648850), bat-CoV/GhanaKwan/19/2008 (FJ710046), bat-CoV/GhanaKwan/20/2008 (FJ710047), bat-CoV/M.das/Germany/D3.3/2007 (EU375854), bat-CoV/USA/RM-11 (EF544563), bat-CoV HKU2 (EF203064), HKU4 (NC_009019), HKU5 (NC_009020), HKU6 (DQ249224), HKU8 (DQ249228), HKU9 (NC_009021), CoV-HKU1 (NC_006577), human coronavirus (HCoV-OC43) (NC_005147), murine hepatitis virus (MHV) (NC_001846), bovine coronavirus (BCoV) (NC_003045), porcine hemagglutinating encephalomyelitis virus (PHEV) (NC_007732), human severe acute respiratory syndrome coronavirus (SARS) (SARS-human) (NC_004718), civet SARS-like coronavirus (SARS-civet) (AY304488), bat-SARS-like coronavirus HKU3 (bat-SARS-CoV HKU3) (NC_009694), infectious bronchitis virus (IBV) (NC_001451), and turkey coronavirus (AF124991).

A 440-bp consensus nt sequence of group 2 CoVs was also obtained (>98% nt identity with each other) and deposited in GenBank as Bat-CoV/Philippines/Diliman1525G2/2008 (DDBJ accession no. AB539081). A BLAST search suggested that the partial RdRp sequence was novel but most similar to that of Bat-CoV/HKU9–1/China/2007 (83% nt identity). A phylogenetic tree was constructed with the partial RdRp-deduced amino acid sequence (120 aa) and available sequences of known CoVs (Figure 1). Data in the tree suggested that Bat-CoV/Philippines/Diliman1552G1/2008 belonged to group 1b CoVs and Bat-CoV/Philippines/Diliman1525G2/2008 belonged to group 2d to CoVs.

Specific and nested primer pairs for group 1b bat CoV and group 2d bat CoV sequences were designed, and nested PCR was performed by using cDNAs of all samples. Twenty additional amplicons (≈200-bp sequences) were obtained by using primers specific for group 2d bat CoVs. After direct sequencing or TA cloning, partial sequences of all amplicons obtained were found to be nearly identical to group 2d bat CoVs (>98% nt identity) and resulted in a total CoV prevalence of 55.8% (Table 1). All sequences of group 1b bat CoVs were obtained from insectivorous bats (4/7, 57.1%), and all sequences of group 2d bat CoVs were obtained from frugivorous bats (25/45, 55.6%).

### Virus in Cell Cultures

Cytopathic effects were not observed in any of the cells (Vero E6, Vero, Hrt18, A549, fcwf-4, BKT-1, Tb-1 Lu, or primary kidney cells derived from Leschenault rousette bats) tested with bat intestinal specimens positive for both detected viruses by RT-PCR. Results of RT-PCR for cell lysates to detect viral replication also were negative.

### Virus Propagation in Fruit Bats

To obtain bat CoVs from field samples, we administered virus orally to 2 Leschenault rousette bats maintained in the Department of Biomedical Science, Graduate School of Agricultural and Life Sciences, University of Tokyo. The volume of intestine samples collected from insectivorous bats was less than that from fruit bats because of their body size, and all positive samples for the group 1b bat CoV genome were derived from small insectivorous bats. Oral infection was conducted only with samples positive for the group 2d bat CoV genome. A homogenized large intestine sample (60 mg) derived from a lesser dog-faced fruit bat, which contained 7.8 × 10^6^ copies of viral genome, was given orally to 2 fruit bats (bats A and B). After confirmation that these bats showed no clinical signs of infection, they were killed 6 days after infection.

Virus genome was detected only in the small and large intestines of both bats by RT-PCR (Table 2). Virus was not detected in these intestine samples by cell cultures. Virus genome was detected by RT-PCR in fecal samples obtained during daily observations for clinical signs, and viral genome copy number was determined by real-time RT-PCR (Table 3). Virus copy number peaked on day 3. On day 4, a fecal sample was not collected because feces were not found on the bottom of the isolator.

**Table 2:** PCR results for bat coronavirus in fruit bats infected by using bat intestinal samples, the Philippines*

Bat	Assay	Liver	Kidney	Lung	Spleen	Brain	Small intestine	Large intestine	Serum
A	RT-PCR	–	–	–	–	–	–	+	–
	qRT-PCR	ND	ND	ND	ND	ND	1.25 × 10^6^	3.53 × 10^6^	ND
B	RT-PCR	–	–	–	–	–	+	+	–
	qRT-PCR	ND	ND	ND	ND	ND	1.47 × 10^6^	1.50 ×10^6^	–

*Bat intestinal samples containing 7.8 × 10^6^ copies of coronavirus genome. Values are copies per milligram. RT-PCR, reverse transcription–PCR; –, virus RNA not detected; +, virus RNA detected; qRT-PCR, quantitative RT-PCR; ND, not done.

**Table 3:** Time course of detection of coronavirus viral genome by PCR in feces from 2 fruit bats, the Philippines*

Test	Days after infection					
	0	1	2	3	4	5
RT-PCR	–	–	–	+	ND	+
Quantitative RT-PCR	–	–	5.31 × 10^4^	1.74 ×10^7^	ND	1.5 × 10^6^

*Values are copies per milligram. RT-PCR, reverse transcription–PCR; –, virus RNA not detected; +, virus RNA detected; ND, not done.

### Virus mRNA in Experimentally Infected Bats

**Figure 2:**
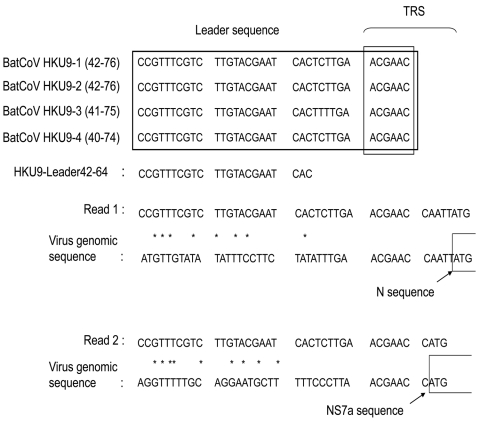
Comparison of mRNA sequences of bat coronavirus (BatCoV) with viral genomic sequences. Read 1 was obtained by using reverse transcription–PCR and HKU9-Leader42–64 and N468–448r primers. Read 2 was obtained by using HKU9-Leader42–64 and Ns7a440–420r primers. Asterisks indicate sequence identity for read and virus genome sequences. TRS, transcription regulatory sequence; N, nucleocapsid; NS, nonstructural.

For murine hepatitis virus and several CoVs, an ≈70-bp leader sequence is added to the 5′ end of the transcription regulatory sequence of each nested mRNA during mRNA processing (*15*,*16*). For bat CoVHKU9–1, which was most similar to group 2d bat CoV, a complete genome sequence and putative transcription regulatory sequence of Bat-CoV HKU9 were predicted (*17*). On the basis of that report, primer HKU9-Leader42–64, including a leader sequence, was designed (Figure 2). The HKU9-Leader42–64 primer and 3siteAd20T primer, which included the oligo dT sequence, were used for PCR with RNA extracted from intestines of bats A and B. Amplicons were cloned, and partial genomic sequences of group 2d bat CoV membrane, nucleocapsid, Ns7a, Ns7b, and Ns7c genes were determined. These sequences were deposited in GenBank (DDBJ accession no. AB543561). A phylogenetic tree was also constructed with the deduced amino acid sequence (463 aa) of the complete N gene of group 2d bat CoV and available sequences of known CoVs. The tree showed the same topology as that constructed with deduced amino acid sequence of the partial RdRP gene. The N gene nucleotide sequence was most similar to that of Bat-CoV/HKU9–1/China/2007 (72% identity).

**Figure 3:**
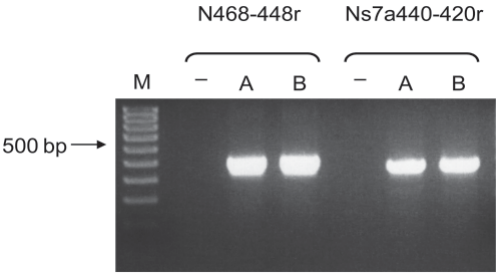
Bat coronavirus/Philippines/Dilliman1525G2/2008 mRNA in experimentally infected fruit bats, the Philippines. Reverse transcription–PCR results for small intestines of bats A and B. Lane M, 100-bp DNA ladder; lane –, nontemplate control.

To confirm presence of transcribed virus mRNA in bats A and B (Table 2), RT-PCR specific for mRNA of the group 2d bat CoVs was conducted with HKU9-Leader42–64, N468–448r, and Ns7a440–420r primers. Virus mRNAs were detected in RNA extracted from the small intestines of bats A and B (Figure 3). All amplicons were sequenced and included the nucleotide sequence of the HKU9-Leader42–64 primer sequence at the 5′ end of the sequences obtained (Figure 2). These results suggest that virus mRNAs were transcribed in bats A and B.

### Experimental Infection of Bats

To determine whether this 2d bat CoV was pathogenic, we experimentally infected 5 *R. leschenaulti* fruit bats. A 60-mg sample of the small intestine from bat A was given orally to 2 bats (bats C and D), and 500 μL of phosphate-buffered saline was given orally to 1 bat (bat E) as a control. These bats were killed 6 days after infection. Clinical signs were not observed in the experimentally infected bats. Virus genome amplification was not detected by RT-PCR in any samples (serum, brain, kidney, liver, lung, spleen, and feces). However, virus RNA was detected in the small intestine (Table 4). No pathologic changes were observed in the intestines or other organs.

**Table 4:** Results of nested and quantitative RT-PCRs of cDNA from bat samples, the Philippines*

Bat	Liver	Kidney	Lung	Spleen	Brain	Small intestine			Large intestine			Serum
C	–	–	–	–	–	+ (ND)			–			–
D	–	+ (ND)	+ (ND)	+ (ND)	–	+ (6.57 × 10^4^)†			–			+ (ND)
E	–	–	–	–	–	–			**–**			**–**
						Intestine section						
						1	2	3	4	5	6	
F	–	–	–	–	–	–	–	+ (ND)	+ (ND)	–	–	–
G	–	–	–	–	–	–	–	+ (ND)	+ (ND)	+ (ND)	+ (5.91 × 10^4^)	–

*cDNA was synthesized from bat samples obtained after experimental infection (second passage of the group 2d coronavirus in Leschenault rousette bats). Values are copies per milligram. Virus load was quantified by real-time PCR. RT-PCR,reverse transcription–PCR; –, virus RNA not detected; +, virus RNA detected; ND, not detected by real-time PCR in RT-PCR–positive samples. †Result of nested PCR.

Because virus growth in fruit bats was weaker than virus growth in bats after primary infection with field samples, experimental conditions were changed. Samples (300 mg) from the small intestine of 2 bats (A and B) were given orally to 2 other bats (F and G), which were killed 3 days after infection. Six intestinal samples were obtained from each bat to determine site specificity of virus growth. The entire intestine (duodenum to the large intestine) was divided into 6 equal parts (1–6). Virus RNA was detected only by RT-nested PCR in the small and large intestines (Table 4), and no pathologic effect was detected in these bats. Virus RNA was detected in the lower region (parts 3–4 from bat F and parts 3–6 from bat G). However, we could not determine the specific site of virus replication.

## Discussion

After the SARS epidemic in 2003, bats were identified as carriers of CoVs in China. Recently, bat CoVs have also been detected in several other regions, including Germany, North and South America, and Africa. In the current study, we confirmed the presence of 2 CoVs in bats in the Philippines. Our findings suggest that CoV circulation in bats is worldwide. Although only 52 bats were tested, CoV RNA was present in 55.8% large intestine samples from these bats. Moreover, all bats tested seemed to be healthy. Thus, bats may be persistently infected carriers of CoVs. These data are consistent with results of previous reports of CoV detection in bats (*3*–*6*).

RNA of group 1b bat CoV was detected in 4 (57.1%) of 7 insectivorous bats. All bats positive for group 1b bat CoV RNA belonged to the same species, the Lesser Asiatic yellow bat (*Scotophilus kuhlii*). However, the partial RdRp sequence of the virus was most similar to that of Bat-CoV/China/A515/2005 (95% nt sequence identity), which was also detected in bats of the same species in the southern China on Hainan Island (*5*). The Lesser Asiatic yellow bat is distributed widely in eastern Asia in Philippines, Pakistan, Hainan Island, Taiwan, and Borneo (*18*). High similarities of sequences between group 1b bat CoV and Bat-CoV/China/A515/2005 suggest that these viruses are distributed widely in bats enzootic to eastern Asia.

Although group 1 bat CoV was detected in 1 species of insectivorous bats, group 2d bat CoV was detected in 4 species of frugivorous bats. Five of the 45 frugivorous bats were positive by RT-PCR, and an additional 20 were positive by RT-nested PCR (prevalence 55.6%). This finding suggests that replication of group 2d bat CoV in the intestine is low.

The complete N sequence of group 2d bat CoV suggests that it is a novel virus and most similar to that of Bat-CoV/HKU9–1/China/2007 (77% aa sequence identity). Woo et al. (*17*) detected Bat-CoV/HKU9–1/China/2007 and classified the viral nucleotide sequence as that of group 2d CoV. Our phylogenetic data (Figure 1) suggest that group 2d bat CoV and Bat-CoV/HKU9–1/China/2007 belong to the same group.

We attempted to isolate bat CoVs from several cell lines and primary cultured cells. However, virus replication was not observed, which is consistent with results of a previous report (*17*). No infectious bat CoV has been isolated from cell culture. In the current study, the amount of large intestine obtained per bat was <100 mg. Therefore, most samples were inadequate for virus isolation, especially virus-positive samples for group 1 bat CoVs from insectivorous bats.

To obtain sufficient tissue to isolate virus RNA, we attempted to infect fruit bats with bat CoV. Although we could not obtain bats of the species from which group 2d bat CoV was detected in the field survey, we obtained Leschenault rousette bats from zoos in Japan. In addition, Bat-CoV/HKU9–1/China/2007, which was most similar to group 2d bat CoVs by phylogenetic analysis, was identified in this species in Hong Kong (*17*). This finding indicates that fruit bats can be infected with this virus. No signs of clinical disease were observed after oral infection with an intestine sample derived from a lesser dog-faced fruit bat. However, virus RNA was detected in the small and large intestines (Table 2), and these intestinal samples contained more genome copies than input copies. Furthermore, virus RNA was amplified in fecal samples by real-time PCR, and viral mRNAs were detected in bats A and B (Figure 3). These findings indicate that group 2d bat CoVs can be orally transmitted to fruit bats and replicate in them.

Experimental infection was conducted in fruit bats by using tissues from virus-infected bats to determine virus pathogenicity. However, infected bats showed no signs of a pathologic effect, although low levels of virus RNAs were detected in the small and large intestines of these bats. These findings suggest that fruit bats can be infected with bat CoV without showing any signs of infection. However, compared with primary infection by field samples obtained from *C. brachyotis*, the level of viral genome amplification was low in experimental infection. This finding may have been caused by a difference in viral replication in bats of different species. In the field survey, partial nucleotide sequences of group 2d bat CoVs, were detected in 4 bat species. A high prevalence of virus RNA was observed in each bat species (Table 1). These findings suggest that the group 2d bat CoVs may infect fruit bats of many species. The oral infection study showed that CoV is easily transmitted across species. These results, and the fact that most reported bat CoV sequences have been detected in several bat species (*12*,*17*), imply that interspecies transmission in bats may be common.

Further investigation of bat CoV ecology is needed to better understand the risk for infection with this virus. Knowing this risk could help elucidate emergence of SARS. Although we demonstrated in vivo propagation of a bat CoV, a bat CoV culture system is needed to obtain additional information about this virus.
